# Foreign language acquisition of perceptually similar segments: evidence from Lower Sorbian

**DOI:** 10.12688/openreseurope.14895.2

**Published:** 2024-02-15

**Authors:** Phil J. Howson

**Affiliations:** 1 Leibniz-Zentrum Allgemeine Sprachwissenschaft, Berlin, 10117, Germany

**Keywords:** Lower Sorbian, sibilant fricatives, language acquisition, phonetics, foreign language acquisition, second language acquisition, ultrasound, endangered languages

## Abstract

Lower Sorbian is a moribund language spoken in Eastern Germany that features a three-way sibilant contrast, /s, ʂ, ɕ/. The vast majority of L1 speakers are above eighty years of age and virtually no young Sorbians learn Lower Sorbian as their first language. There are language revitalization programs in place, but this means that virtually all Lower Sorbian speakers are L2 learners whose first language is German. German, as opposed to Lower Sorbian, has a two-way sibilant contrast, /s, ʃ/. So, Lower Sorbian learners need to acquire a perceptually similar sibilant contrast, /ʂ, ɕ/, that commonly assimilates with a single L1 segment, /ʃ/. The two-to-one assimilation makes acquisition difficult. In this project, I examine the acquisition of the three-way sibilant contrast using ultrasound technology. The ultrasound data revealed that learners in the contemporary context do not produce a distinction between /ʂ, ɕ/ and only learners at an advanced level who had significant exposure to L1 speakers have acquired a three-way sibilant distinction. The findings are put into the context of models of L2 acquisition and generalized implications for foreign language acquisition are discussed.

## Plain language summary

Second language acquisition requires that language learners acquire a novel set of speech segments. For young Sorbians who learn Lower Sorbian as a second language, they must acquire two novel sibilant fricative segments (high frequency noisy segments like /s/). Both of these segments are perceptually similar to the German sibilant fricative common represented with *sch* (e.g., Schlange *snake*). This study explores the acquisition of the Lower Sorbian sibilant fricative contrasts using ultrasound technology. Ultrasound records video of tongue contours at a high-frame rate so that statistical analysis of tongue shapes can be performed. In this project, I examine the tongue contours for Lower Sorbian learners at the beginner, intermediate, and advanced levels of acquisition to observe how tongue shapes for sibilant fricatives are acquired. The results implicate that in a language revitalization context where few L1 speakers are available, the input that learners receive should be augmented with pronunciation and perceptual resources to assist in acquisition. Specific recommendations are provided.

Auf Deutsch

Der Zweitspracherwerb setzt voraus, dass die Sprachlernenden eine Reihe neuer Sprachsegmente erwerben. Junge Sorben, die Niedersorbisch als Zweitsprache lernen, müssen zwei neue Zischlaute (hochfrequente, laute Segmente wie /s/) erwerben. Diese beiden Segmente ähneln in der Wahrnehmung dem deutschen Zischlaut, der häufig mit sch dargestellt wird (z.B. Schlange). In dieser Studie wird der Erwerb der niedersorbischen Zischlautkontraste mittels Ultraschalltechnologie untersucht. Ultraschall zeichnet Videos von Zungenkonturen mit einer hohen Bildrate auf, so dass eine statistische Analyse der Zungenformen durchgeführt werden kann. In diesem Projekt untersuche ich die Zungenkonturen niedersorbischer Lerner auf der Anfänger-, Mittelstufen- und Fortgeschrittenenstufe, um zu beobachten, wie Zungenformen für Zischlaute erworben werden. Die Ergebnisse deuten darauf hin, dass in einem Sprachwiederbelebungskontext, in dem nur wenige L1-Sprecher zur Verfügung stehen, der Input, den die Lernenden erhalten, mit Aussprache- und Wahrnehmungsressourcen ergänzt werden sollte, um den Erwerb zu unterstützen. Es werden spezifische Empfehlungen gegeben.

## Introduction

Lower Sorbian is a west Slavic language spoken in Eastern Germany. It is a moribund language ( Moseley, 2012) and is spoken near the border of Poland ( Stone, 1993). The vast majority of first language Lower Sorbian speakers are above 80 years of age. Additionally complicating the matter, is that the language situation in Lower Sorbian is quite precarious. The majority of first language speakers do not use their mother tongue in daily communication which has led to certain degrees of language attrition. Additionally, nearly every young speaker of Lower Sorbian is a second language learner and acquires the language at school. For example, the *Witaj* program is a kindergarten curriculum which incorporates Lower Sorbian into the students’ education. Following that, many students participate in the *Dolnoserbski gymnazium Chóśebuz*, situated in Cottbus ( Marti, 2007). The school completes up to grade 12 and includes Lower Sorbian as a mandatory aspect of education. While the education can be beneficial, there is difficulty finding qualified teachers for the school and due to the advanced age of the L1 speakers, teachers are typically second language speakers themselves.

Lower Sorbian has a cross-linguistically uncommon three-way contrast among sibilant fricatives (approximately less than 6% of languages in the world; Maddieson, 1984) that makes contrasts at the dental/alveolar, /s, z/, retroflex, /ʂ, ʐ/, and alveolopalatal, /ɕ, ʑ/, places of articulation, similar to the contrasts observed in Modern Polish ( Żygis, 2003). The contrast contains two sibilants, /ʂ, ɕ/, that share acoustic-perceptual similarities to /ʃ/. Many theories of language acquisition, such as the PAM-L2 ( Best & Tyler, 2007) and the SLM-r ( Flege & Bohn, 2021), have postulated how different aspects of acoustic-perceptual similarities with L1 segments impacts L2 acquisition. Contrasts such as the three-way contrast, under these theories are the most difficult to acquire due to the acoustic similarities between the segments. This makes Lower Sorbian an excellent language to examine foreign language acquisition of sibilant fricatives.

## Second language acquisition

### The PAM-L2

The Perceptual Assimilation Model of L2 Acquisition (PAM-L2; Best & Tyler, 2007) is an extension of the Perceptual Assimilation Model (PAM; Best, 1995) to second language acquisition. The PAM-L2 is a direct realist model, which assumes that perception is related to the perception of distal articulatory events (i.e., changes in vocal tract configurations), not specific acoustic patterns. Under the view of the PAM-L2, perceptual learning can take place on multiple levels, including phonological, phonetic, or gestural. One way in which category acquisition can occur is when there are two L2 segments that assimilate to two separate L1 segments (two-category assimilation). The PAM-L2 predicts good to excellent discrimination in this context. Learners then continue to acquire L2 vocabulary using the assimilated categories. This leads to a common L1-L2 phonological category for each of the L2 segments. However, in the case that there is a perceptible phonetic difference between L1-L2 pairs of segments, then it is possible that this difference becomes perceptibly stronger for the learner with time. If the differences between L1-L2 pairs becomes perceptible enough, then separate L1 and L2 phonetic categories can emerge. However, if the distinction is not strong enough the learner will not develop separate L2 categories ( Tyler, 2019). This process is assumed to occur very early in acquisition, although it may strengthen over time.

Best *et al.* (2009) suggest the process of perceptual attunement is tightly related to vocabulary acquisition. Bundgaard-Nielsen *et al.*’s (2012) *Vocabulary-Tuning Model of L2 Rephonologization* posits that an increase in vocabulary size drives perceptual attunement to L2 phonological structure. Support for this position was found by Bundgaard-Nielsen, Best, & Tayler ( 2011a, 2011b); however, Tyler (2019) suggests that an increase in vocabulary might support the acquisition of more discriminable L1-L2 pairs but could inhibit less discriminable pairs. Thus, Tyler (2019) suggests that the opportunity for phonetic learning is likely before the L2 vocabulary exceeds 50 words. He supports this position by comparing this to cL1 acquisition; children slow their vocabulary up to around 50 words, and then a rapid increase in vocabulary occurs after (e.g., Nazzi & Bertoncini, 2003). For Tyler (2019), after phonetic attunement takes place, vocabulary increase ramps up dramatically. Thus, the effect of learning a large vocabulary prior to phonetic attunement of difficult to perceive contrasts greatly hinders acquisition.

In the case of Lower Sorbian acquisition, there are two segments of interest that are perceptually similar, /ʂ, ɕ/, which are both perceptually similar to the same L1 segment, /ʃ/. According to the PAM-L2, this is single category assimilation and poor discrimination is predicted. Although, there may still be relative goodness-of-fit difference between the two assimilatory segments that allows learners to discriminate between them and thus acquire the L2 segments. However, the PAM-L2 and its predictions focus on learners in an immersion environment (second language acquisition; SLA); learning a second language in the learner's L1 environments with L2 classes (foreign language acquisition; FLA) has differences from immersion learning ( Tyler, 2019). Nonetheless, the PAM-L2 can offer potential insights into foreign language acquisition (FLA). Tyler (2019) suggests that single-category assimilations (i.e., two L2 segments assimilating to the same L1 segment) are even more unlikely to be acquired in the classroom setting. The reason for this is because of a reduced access to consistent stimuli and the phonetic contrasts that distinguish them. Many second language classrooms are also taught by second language speakers, who may or may not consistently produce the language relevant contrasts, and likely produce contrasts differently than the older generation of L1 speakers. Additionally, there is also extensive acoustic-perceptual input from other second language learners, who also may not produce a target contrast. Tyler (2019) also notes that there is an increase in how fast vocabulary is acquired relative to immersion and L1 contexts which could impact perceptual acquisition.

### The Speech Learning Model

The speech learning model (SLM; Flege, 1995) and the revised speech learning model (SLM-r; Flege & Bohn, 2021) have also been frontrunners of second language acquisition theories. The SLM was primarily designed to account for age related differences in language acquisition, while the SLM-r aims at providing an explanation for how reorganization of the phonetic system occurs over the life-span due to naturalistic L2 learning.

The SLM posits that for late acquiring bilinguals (i.e., someone who acquired two languages as a child, but the second language was acquired later than the first), L2 phonetic learning is influenced by acoustic-perceptual similarities between L2 and L1 phonetics. Thus, L1 and L2 segments become perceptually linked together. Specifically, during L2 learning, segments “map onto” perceptually similar L1 sounds. The ability for L2 learners to discern perceptually linked sounds occurs gradually, rather than rapidly; however, when this occurs, formation of a novel phonetic category can occur.

The mechanisms for novel category formation that guide L1 acquisition are believed to be intact and available for L2 learning. In L1 acquisition, this process is slow and begins as a set of equivalence classes ( Kuhl, 1983) that involves grouping acoustically similar sounds together. This development continues long after establishing a phonetic inventory ( Lee *et al.*, 1999) and extends at least beyond the age of seven years ( Bent, 2014). The SLM proposes that L2 learners of any age form acoustic-perceptual equivalence classes from the statistical properties of the input distributions of their exposure to the target L2. However, unlike L1 category formation, which has no previous language exposure and categories to interfere with it, L2 category formation relies on disruption of L2-to-L1 perceptual links through the ability to discern phonetic differences between perceptually similar L2 and L1 segments. Flege & Bohn (2021) suggest that L2 category formation should take at least as long as L1 category formation.

According to the SLM, L2 category formation depends on the degree of acoustic-perceptual similarity between the L2 segment and the closest L1 sound. That is, the more similar it is to an L1 segment, the harder it will be to form a new L2 category. Additionally, age of acquisition plays a significant role, with older learners having lower probabilities of forming new categories.

The SLM-r ( Flege & Bohn, 2021) maintains that there is no difference in how L2 segments are acquired compared to L1 acquisition. The SLM-r posits that observed differences in L2 acquisition, and subsequently, the production and perception of L2 segments arise because L2 sounds are initially linked to L1 segments and serve as a substitute, especially for early learning. The existing L1 phonetic categories interfere with and can even block the formation of novel categories as a result. Additionally, L2 acquisition typically has a different set of input stimulus, which often includes foreign accented L2 speech.

The SLM-r distinguishes itself from the PAM-L2 in that it posits that the delinking process can be facilitated by growth of an L2 lexicon ( Bundgaard-Nielsen *et al.*, 2011a; Bundgaard-Nielsen *et al.*, 2011b). While the PAM-L2 believes that growth of the L2 lexicon (beyond perhaps ~50 words) serves to stagnate L2 category formation, at least in the case of hard to discriminate L1 and L2 segments ( Tyler, 2019). In this sense, the SLM-r puts forth that category formation is a much longer and drawn-out process ( Flege & Bohn, 2021), while the PAM-L2 suggests it is a quicker process with a narrow opportunity for learners to acquire a new category ( Tyler, 2019). Additionally, the PAM-L2 posits that learners attenuating to gestural movements in the vocal tract, while the SLM-r suggests that learners pay attention to acoustic differences in the input signal directly. Thus, under the view of the SLM-r, articulation is a matter of better navigation of what vocal tract shapes produce the target acoustic outputs.

### Hypothesis

Based on both the PAM-L2 and SLM-r, the anticipated patterns of L2 segment assimilation is that learners will assimilate both Lower Sorbian, /ʂ, ɕ/, to German /ʃ/. This is due to the acoustic-perceptual similarities between them. The acoustics between the two segments /ʂ, ɕ/ resemble each other across in COG and skewness, having both a lower COG and higher skewness than /s/. Both values also significantly overlapped with each other for /ʂ, ɕ/. The feature in Lower Sorbian that was found to most strongly distinguish /ʂ, ɕ/ from each other was a much higher transitional F2 into the following vowel for /ɕ/ compared to /ʂ/ ( Howson, 2015). The lower COG values observed in Lower Sorbian, tend to match cross-linguistic COG associated with /ʃ/ ( Żygis, 2010) and COG and skewness measures associated with German /ʃ/ ( Weirich & Simpson, 2015). Thus, I expect that low level (i.e., A-level) learners will share tongue contours for /ʂ, ɕ/ and that they will both resemble /ʃ/. It remains possible that there are still goodness-of-fit (or phonetically discernible) differences between /ʂ, ɕ/ and /ʃ/. More specifically, /ɕ/ has formant transitions and spectral characteristics similar to /ʃ/, while /ʂ/ has similar spectral characteristics, but different formant transitions. Thus, I expect that more advanced learners of Lower Sorbian will initially differentiate /ʂ/ from /ɕ, ʃ/ because of the stronger acoustic-perceptual dissimilarities. In terms of the PAM-L2 ( Best & Tyler, 2007), the assumption is that learners are perceiving articulatory gestures and vocal tract changes, not more abstract acoustic characteristics. The implication of this is that as learners become more advanced, they become better at retrieving the articulatory movements necessary to produce a contrast. The expectation is that gradual improvement in the articulation of L2 segments should occur. In terms of the SLM-r ( Flege & Bohn, 2021), there is a similar expectation. As learners’ acoustic-perceptual representation improves, so too should articulation. However, because perceptual (and articulatory) dissimilarities may take more time to pick up on ( Flege & Bohn, 2021), I predict that only more advanced learners will have acquired these contrasts.

## Methods

### Study design

The study design was an articulatory examination of tongue contours using ultrasound data collection techniques. Participants read sentences in Lower Sorbian with the target segments in them while they were being recorded with ultrasound. Tongue contours were compared using Generalized Additive Mixed Models (GAMMs). Data recording took place from March 27 ^th^, 2020 until April 1 ^st^, 2020 in Cottbus, Germany for the L2 learners. The advanced L2 learners, C04 and C05, were recorded at the University of Leipzig in Germany from April 4 ^th^ until April 8 ^th^. The L1 speakers were recorded from July 18 ^th^, 2022 until July 22 ^nd^, 2022 in Cottbus, Germany.

### Participants

As a baseline, 1 bilingual Sorbian/German speaker (male, 24), and 1 late-acquiring bilingual speaker of Sorbian (female, 40; age of first acquisition: 5) were recorded using ultrasound. These participants were chosen for this study because they both had significant input stimuli during the learning process from L1 speakers. Both speakers had input from L1 speaking relatives and additionally the older speaker attended the Sorbian school at a time when L1 Sorbian speaking teachers were active. Additionally, at the time of recording this data, few L1 speakers remain, and the advanced age of potential participants (above 80 years of age) makes ultrasound data especially difficult to record and interpret.

The criteria for language learner selection were that participants attended *Dolnoserbski gymnazium Chóśebuz* in Cottbus and were currently engaged in their language learning program. All participants had a first language of German. Participants were recruited for all three skill levels, A-, B-, and C-level learners based on a scaling system like the CEFR. Their skill level at the time was based on class they attended for Sorbian language at *gymnazium Chóśebuz* at the time of recording. Year learning Lower Sorbian ranged from approximately 6–17 years and was not necessarily reflective of the level of the speaker (i.e., more years did not necessarily reflect higher proficiency). Participant saturation was determined based on typical sample sizes for ultrasound studies. For baseline speakers, participants were selected on the basis that they had early exposure to Lower Sorbian and learned it in a natural setting (i.e., through hearing Lower Sorbian), although both participants also received an education in the Lower Sorbian school system. The Lower Sorbian speaking community is small, especially with respect to L1 speakers and so as many L1 speakers as possible were recruited. The L2 learners consisted of 4 A-level, 6 B-level, and 5 C-level learners. All of these participants were ages 17-18. Two of the C-level speakers were extremely advanced. Their ages were 35 and 56. All participants had no self-reported history of speech or hearing disorders.

### Procedure

All participants read and signed the ethics forms prior to the experiment. They were also verbally informed as to the structure of the experiment and informed of their primary rights as a participant, including that their de-identified data would be shared with other researchers, and that they could refuse data sharing if they wished.

Data for the baseline speakers were recorded in a quiet room in the *Serbski Institut* in Cottbus, Brandenburg. Data for the Lower Sorbian learners were recorded in a quiet room at *Dolnoserbski gymnazium Chóśebuz* in Cottbus, Brandenburg. Ultrasound data were recorded with the Micro system from Articulate Assistant Advanced (AAA). I used the 20mm Radius probe with a 92 degrees field of view (FOV). Data was recorded at an average of 80 frames per second (fps). An ultrasound stabilization headset ( Articulate Instruments Ltd., 2008) was also used to prevent movement of the ultrasound probe.

Participant forms were filled out prior to participation, including the questionnaire and consent forms. In order to pseudo-anonymize participant data, participants were assigned a letter and number combination which corresponded to their skill level and the order in which they participated (e.g., C05 = the fifth C-level learner recorded; LS01 = the first L1 Lower Sorbian speaker recorded). Stimuli were presented using the AAA software package. Additionally, audio and video were synchronized and recorded using the AAA software. The full stimuli list is presented in Table 1. Stimuli were presented in a carrier phrase to facilitate more natural production. The carrier phrase was “Grońśo *target* hyšći raz” (please *target* say again). Stimuli were presented in a pseudorandomized order. Each participant produced 6 articulations of each segment in each of the three vocalic environments. This gives a total of 108 tokens for the L1 speakers (2 speakers × 3 segments × 3 vowels × 6 repetitions), 216 tokens for the A-level learners (4 speakers × 3 segments × 3 vowels × 6 repetitions), 324 tokens for the B-level learners (6 speakers × 3 segments × 3 vowels × 6 repetitions), and 270 tokens for the C-level (5 speakers × 3 segments × 3 vowels × 6 repetitions).

**Table 1.** Stimuli.

	*i*		*a*		*u*	
s	ćis	*yew tree*	cas	*time*	kus	*bite*
ʂ	liš	*excessive*	praš	*leprosy*	duš	*soul*
ɕ	biś	*beat*	braś	*take*	duś	*beat*

### Ethical considerations

Ethical approval was obtained from the Deutsche Gesellschaft für Sprachwissenschaft (DGfS #2021-13-220106) and informed written consent was obtained from all participants for the use and publication of their data.

### Analysis

Tongue contours were manually traced using AAA software (v220.04.01) at the temporal midpoint of the fricative. The midpoint was identified based on the duration of the fricative, where the onset was measured as the offset of formants and periodic sound waves associated with the preceding vowel and the offset was determined as the reduction in aperiodic noise and dissipation of frication on the spectrogram associated with the fricative. Polar coordinates were then extracted. Tongue contours were then compared using a custom script ( Heyne *et al.*, 2019) for GAMM analysis of polar coordinates in R ( R Core Team, 2023). GAMMs were performed using the mgcv package ( Wood, 2011), which also provides summary statistics. Tongue contours were first compared for L1 speakers to provide a baseline for comparison. Tongue contours were then compared for each language group (A, B, and C). Group C was split into two: C-level and highly advanced C-level. GAMMs were performed with parametric fixed effects for segment (3 levels: /s, ʂ, ɕ/) and environment (3 levels: /i, a, u/). The interaction between segment and environment was also included. A smoothing variable was also included for segment and the interaction between segment and environment. I included a factor smooth (i.e., a random effect) for the interaction between segment and speaker. The dependent variable was r, or the angle of the coordinate from the probe origin, and each smooth included Theta, which is the distance of the coordinate from the probe origin. For all smooths, cubic regression was used. The equation I used is printed in (1).

     (1) r ~ Segment * Environment + s(Theta, bs = “cr”, k = 25) + s(Theta, by = Segment, bs = “cr”, k = 25) + s(Theta, by = Segment : Environment, bs = “cr”, k = 25) + s(Theta, by = Segment : Speaker, bs = “fs”, k = 25, m = 1)

I also performed an individual analysis for each speaker, which includes a factor smooth for repetition. Because of differences in speaker tongue sizes, k (knots) was set to 20 in order to maintain consistency across all speakers. The equation is printed in (2).

     (2) r ~ Segment * Environment + s(Theta, bs = “cr”, k = 20) + s(Theta, by = Segment, bs = “cr”, k = 20) + s(Theta, by = Segment : Environment, bs = “cr”, k = 20) + s(Theta, by = Rep, bs = “fs”, k = 20, m = 1)

Data was then visualized with plotly ( Sievert, 2020) and a custom script ( Heyne *et al.*, 2019) to identify areas of statistical significance.

## Results

### L1 Speakers

**Figure 1.**
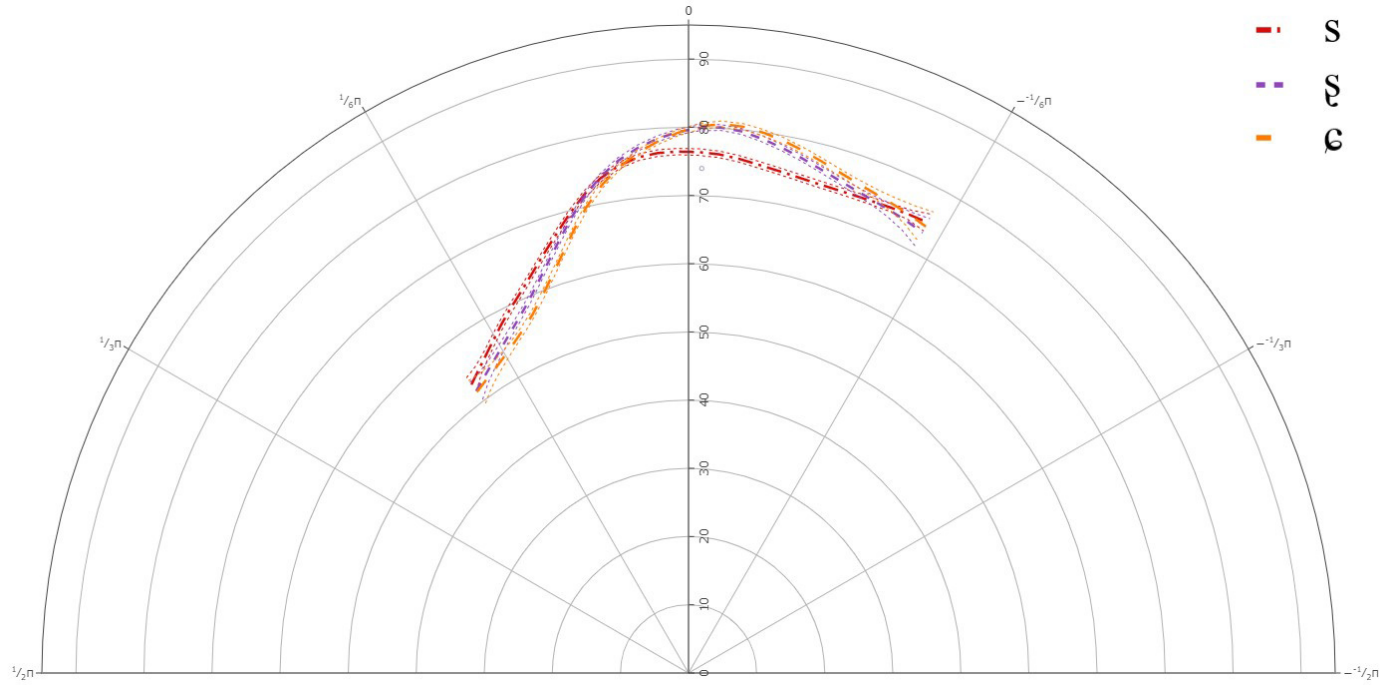
Tongue contours LS Speaker LS101 for /s/ (red), /ʂ/ (purple), and /ɕ/ (yellow).

**Figure 2.**
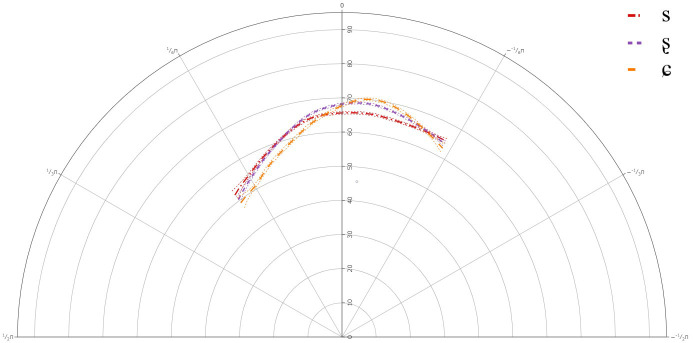
Tongue contours LS Speaker LS102 for /s/ (red), /ʂ/ (purple), and /ɕ/ (yellow).

Figure 1– Figure 2 below present the GAMM smooths for the L1 speakers of Lower Sorbian and Table 2– Table 3 present the approximate significance for the interaction between theta and segment. For full statistical print-outs, see *Extended dat* *a* ( Howson, 2023). The adjusted R ^2^ for the models were 0.979 and 0.983.

**Table 2.** Approximate significance of smoothing term Theta by Segment for L101.

	edf	ref.df	F	p-value
s(Theta)	12.20	14.10	140.821	< 0.001
s(Theta): /s/	7.38	9.09	8.039	< 0.001
s(Theta): /ʂ/	1	1	1.399	0.237
s(Theta): /ɕ/	3.59	4.73	3.404	0.009

**Table 3.** Approximate significance of smoothing term Theta by Segment for L102 speakers.

	edf	ref.df	F	p-value
s(Theta)	9.388	10.967	239.21	< 0.001
s(Theta): /s/	2.835	3.532	104.195	< 0.001
s(Theta): /ʂ/	1	1	109.831	< 0.001
s(Theta): /ɕ/	4.04	5.103	1.436	0.1879

The GAMMs for the L1 speakers revealed a significant difference between all three segments, /s, ʂ, ɕ/. The tongue dorsum was most retracted for /s, ʂ/ and was more advanced for /ɕ/. The tongue contours for /s, ʂ/ were similar, but the tongue body was more raised for /ʂ/. /ɕ/ had the most raised tongue body, but it was not much more raised than /ʂ/.

### A-Level learners

**Figure 3.**
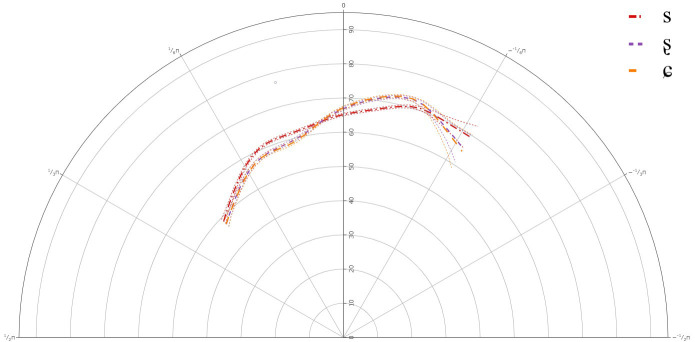
Tongue contours for A-level learners for /s/ (red), /ʂ/ (purple), and /ɕ/ (yellow).

Figure 3 below presents the GAMM smooths for A-level learners of Lower Sorbian and Table 4 presents the approximate significance for the interaction between theta and segment. For individual plots and full statistical print-out, see *Extended dat* *a* ( Howson, 2023). The adjusted R ^2^ for the model was 0.946.

**Table 4.** Approximate significance of smoothing term Theta by Segment for A-level learners.

	edf	ref.df	F	p-value
s(Theta)	11.90	14.288	17.053	< 0.001
s(Theta): /s/	1	1	8.995	0.003
s(Theta): /ʂ/	1	1	0.264	0.607
s(Theta): /ɕ/	4.29	5.45	1.829	0.094

The general results for the A-level learners revealed that there was a significant difference between /s/ and /ʂ, ɕ/, but not between /ʂ/ and /ɕ/. This suggests that learners at the A-level share one tongue contour for their pronunciations of /ʂ, ɕ/. The general tongue contours indicated a more retracted tongue dorsum for /s/, than for /ʂ, ɕ/. The contours for /ʂ, ɕ/ had a slightly more advanced dorsum, with a raised tongue body, resembling /ʃ/, which is present in the L1 German.

Individual results revealed significant deviations in learners’ articulation of /ʂ, ɕ/, when compared against the general tongue contour from the group level GAMM. Although it should be noted that none of the individual plots revealed that any of the learners had acquired the three-way contrast, there was significant variation in their articulation of /ʂ, ɕ/.

### B-Level learners

**Figure 4.**
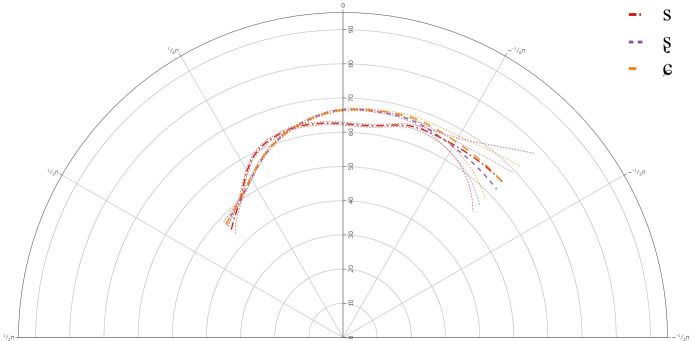
Tongue contours for B-level learners for /s/ (red), /ʂ/ (purple), and /ɕ/ (yellow).

Figure 4 below presents the GAMM smooths for B-level learners of Lower Sorbian and Table 5 presents the approximate significance for the interaction between theta and segment. For individual plots and full statistical print-out, see *Extended dat* *a* ( Howson, 2023). The adjusted R ^2^ for the model was 0.957.

**Table 5.** Approximate significance of smoothing term Theta by Segment for B-level learners.

	edf	ref.df	F	p-value
s(Theta)	10.54	12.59	424.179	< 0.001
s(Theta): /s/	0	0	0.07	0.997
s(Theta): /ʂ/	1.43	1.714	0.197	0.738
s(Theta): /ɕ/	1	1	0.01	0.919

The GAMM results indicated that there was a significant difference between /s/ and /ʂ, ɕ/, but not between /ʂ/ and /ɕ/. This suggests that like the A-level learners, the B-level learners also have not acquired the three-way contrast between /s, ʂ, ɕ/ with respect to their articulation. The general tongue contours reveal a more retracted tongue dorsum for /s/, with a lower tongue body than /ʂ, ɕ/. The contours for /ʂ, ɕ/ had more rounded tongue shape, with more fronting, and more posterior tongue body raising than for /s/.

Individual results also revealed variation in the articulation of /ʂ, ɕ/, although as with the A-level learners, there were no significant differences between /ʂ, ɕ/. In most cases, the tongue dorsum was more drawn back for /s/ and was more advanced for /ʂ, ɕ/. In some cases, the more anterior part of the tongue body was raised for /ʂ, ɕ/, while for some learners the more posterior part of the tongue body or the entire tongue body for /ʂ, ɕ/ was more raised than /s/. This suggests that learners at the B-level continue to use the same segment in place for both /ʂ, ɕ/, although there was a great deal of variation in its realization.

### C-Level learners

**Figure 5.**
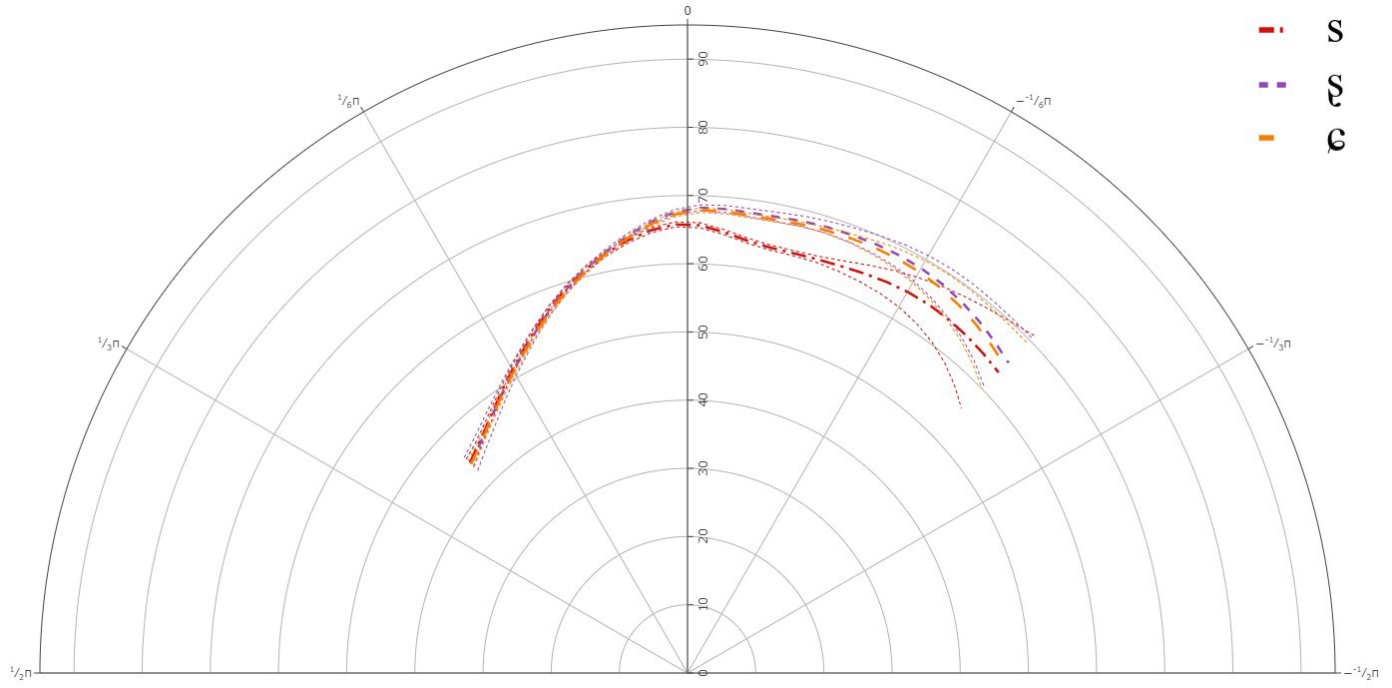
Tongue contours for C-level learners for /s/ (red), /ʂ/ (purple), and /ɕ/ (yellow).

Figure 5 below presents the GAMM smooths for C-level learners of Lower Sorbian and Table 6 presents the approximate significance for the interaction between theta and segment. For individual plots and full statistical print-out, see *Extended dat* *a* ( Howson, 2023). The adjusted R ^2^ for the model was 0.979.

**Table 6.** Approximate significance of smoothing term Theta by Segment for C-level learners.

	edf	ref.df	F	p-value
s(Theta)	12.220	14.380	78.058	< 0.001
s(Theta): /s/	6.516	7.923	9.604	< 0.001
s(Theta): /ʂ/	0	0	0.360	0.994
s(Theta): /ɕ/	1	1	0.042	0.838

The GAMM results for C-level learners indicated that there was a significant difference between /s/ and /ʂ, ɕ/, but not between /ʂ/ and /ɕ/. This suggests that learners of Lower Sorbian at all levels have not acquired the three-way contrast. The individual results showed variation in articulation between speakers and in the case of the C-level learners, none of them showed the same backing of the tongue dorsum for /s/ compared to /ʂ/ and /ɕ/.

### Highly advanced C-Level learners

**Figure 6.**
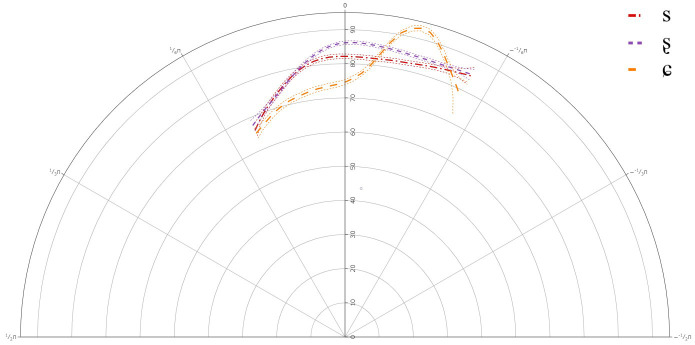
Tongue contours for C04 for /s/ (red), /ʂ/ (purple), and /ɕ/ (yellow).

**Figure 7.**
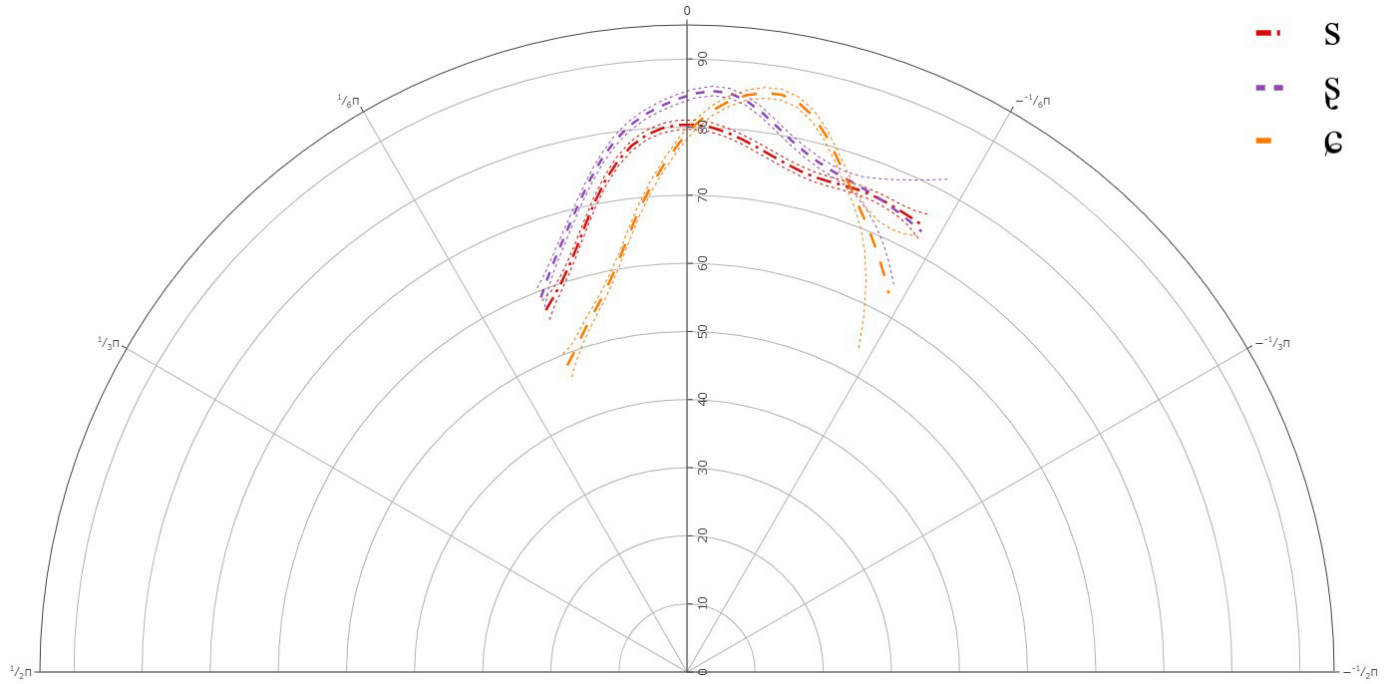
Tongue contours for C05 for /s/ (red), /ʂ/ (purple), and /ɕ/ (yellow).

Figure 6 and Figure 7 below presents the GAMM smooths for highly advanced C-level learners of Lower Sorbian and Table 7 and Table 8 presents the approximate significance for the interaction between theta and segment. The adjusted R ^2^ for the models were 0.970 and 0.972, respectively. The *Extended data* ( Howson, 2023) presents the full statistical printouts of both models.

**Table 7.** Approximate significance of smoothing term Theta by Segment for C04.

	edf	ref.df	F	p-value
s(Theta)	8.78	10.45	67.019	< 0.001
s(Theta): /s/	0	0	0.385	0.997
s(Theta): /ʂ/	1	1	0.116	0.734
s(Theta): /ɕ/	11.90	13.98	47.547	< 0.001

**Table 8.** Approximate significance of smoothing term Theta by Segment for C05.

	edf	ref.df	F	p-value
s(Theta)	8.618	10.029	4.493	< 0.001
s(Theta): /s/	5.127	6.194	1.851	0.087
s(Theta): /ʂ/	3.495	4.184	2.407	0.0475
s(Theta): /ɕ/	8.284	10.099	8.737	< 0.001

In both cases, the learners acquired a three-way contrast for /s, ʂ, ɕ/; however, the realization of /ʂ, ɕ/ varied for both speakers. In both cases, /s/ had the lowest tongue body, accompanied by retracted tongue dorsum. /ʂ/ for C04 had a similar degree of retraction for the tongue dorsum as /s/, with a more raised tongue body. The tongue shape for /ʂ/ was faithful to the L1 pronunciation. /ɕ/ for C04 had a low and advanced tongue dorsum. This shape is likely due to the high degree of anterior tongue body advancement and raising. This tongue shape deviated significantly from the L1 pronunciation for /ɕ/. /ʂ/ for C05 had even more tongue dorsum retraction than /s/, with a raised posterior tongue body that had a downward sloping anterior tongue body. /ɕ/ for C05 had a more advanced tongue dorsum and tongue body. The posterior tongue body was raised, with a downward sloping anterior tongue body.

## Discussion

The analysis revealed that for L1 speakers, there is a 3-way contrast intact, but that for L2 learners, substitution of both /ʂ, ɕ/ for /ʃ/ occurred even for learners at the C-level. This was true for all learners except the most highly advanced C-level speakers. Both the PAM-L2 and SLM-r predict that such an assimilation would occur and that the contrast should be difficult to acquire because of the acoustic-perceptual similarity between the two. Nevertheless, in an immersion context, both models predict that it is possible for learners to acquire these contrasts. However, the observed learners were in a foreign-language context and the educators were primarily second language learners themselves. This means that there was likely varied input and the lack of access to L1 input may have greatly hindered their acquisition. However, it should be noted that one limitation of the study is the relatively small wordlist which makes it more difficult to assess category formation.

Learners in this dataset were not given specific pronunciation instructions. What this means is that learners only had access to any existing internal language learning mechanisms. Flege (1995) predicts that the mechanisms involved in L1 acquisition are still available for L2 learners and the evidence presented here does not disprove this but, at the least, it suggests that L1 interference in the acoustic-perceptual space ( Kuhl, 1991; Kuhl *et al.*, 1992; Kuhl & Iverson, 1995) significantly interferes with language learning mechanisms if they are still accessible. The result is that the distortion of the perceptual space inhibits perceptual learning of L1 assimilated segments and thus hinders any alteration in articulatory patterns and novel category formation. As a result, learners have a merging between /ʂ, ɕ/ in Lower Sorbian into their L1 German /ʃ/ category. One caveat to note is that the current L2 instructors do not have the level of fluency as the L1 instructors that the two advanced speakers (C04 and C05) had access to. As such, it is difficult to interpret how much input for the three-way sibilant contrast (if any) learners received. It is clear from discussions with the learners that pronunciation lessons are not a regular part of the curriculum. It remains very possible the lack of acquisition of the three-way contrast is predominantly due to lack of the three-way contrast in the input for learners. In short, the development of the language in the context of endangerment, revitalization, and its status as a minority language in the German context has possibly led to an inventory shift away from a three-way contrast to a more typical two-way contrast like the one observed in Upper Sorbian ( Howson, 2017). If the desire of the community is to maintain specific speech patterns present in older L1 speakers, then from a practical standpoint, it seems that additional resources need to be committed to this achieve this goal. This is at least true in the foreign language context but would undoubtably assist in immersion contexts as well. Idealistically, this would involve perceptual training that would cater to the speaker’s L1 segments and assist in training the learner in distinguishing their existing L1 categories and L2 categories. This would also be accompanied by specific instructions on how the target segments are produced. Ultrasound technology has been used in this context both for direct visualization of how the learner produces the contrast themselves and how they should produce the contrasts ( Antolík *et al.*, 2019) as well as providing visual instruction guides for learners ( Bliss *et al.*, 2018). This indicates that in language learning and preservations efforts, a multitude of resources should be employed to assist second language learners in acquisition of L2 segments.

There is also the case of the two highly advanced speakers who have acquired a three-way contrast in their L2 speech. First and foremost, the speakers are much older, and as a result had a significant amount of input from L1 speakers during their acquisition processes. The increased access to authentic speech could have contributed to the eventual formation of novel categories. However, it is also important to note that Lower Sorbian /ɕ/ for both speakers appears to have been assimilated into the German /ʃ/ category. In terms of the PAM-L2, this would suggest a better goodness-of-fit match between /ɕ/ and /ʃ/. While, /ʂ/ has similar spectral qualities, the formant transitions are much more similar between /ɕ/ and /ʃ/, while also having similar spectral qualities. This suggests that at least a certain degree of perceptual dissimilarity must be present for the acquisition process to take place. When a segment is “good enough,” rather than forming a novel category, the L1 category becomes linked (in SLM terms). Whether or not L1 phonological patterns are imported into L2 or if L2 influences L1 phonological patterns is unclear. Additionally, it remains unclear if phonetic linking occurs with a decoupling of phonological behaviour. As a result, the interaction in phonological patterning and effects between L1 and L2 linked segments needs to be explored further.

## Data Availability

OSF: L2 Lower Sorbian. https://doi.org/10.17605/OSF.IO/DAURS. ( Howson, 2023) This project contains the following underlying data: lower_sorbian_dataset.xlsx (data used in the statistical analyses.) participant_data.pdf (data from the participant questionnaires.) This project contains the following extended data: extended_data_for_Howson_2023.pdf (full statistical print outs and plots for all the models presented in this paper.)
