# Supplementary material for: Foreign language acquisition of perceptually similar segments: evidence from Lower Sorbian
Source: Open Res Eur. 2024 Feb 15;3:56. Originally published 2023 Apr 13. [Version 2] doi: 10.12688/openreseurope.14895.2 (PMC10964000; doi:10.12688/openreseurope.14895.2)
Supplement: Peer review history

# Reviewer report: Reviewer response for version 2

**DOI:** 10.21956/openreseurope.18664.r38172

**Published:** 2024-03-25

**Authors:** Claire Nance

**Affiliations:** 1 Linguistics and English Language, Lancaster University, Lancaster, England, UK

The author has now addressed our main points.

 I would still recommend making the following minor changes:

 I still find the distinction between 'L1 speakers' and 'baseline speakers' confusing I'm afraid. It is clear that it would not be possible to ultrasound 80+ year old L1 speakers. My understanding is that the 'baseline' speakers in this study are sequential bilinguals who have achieved very high proficiency in Sorbian, with lots of L1 Sorbian input. That's completely fine. But it's confusing to refer to them as 'L1 speakers'. Suggest changing to 'Baseline speakers' or similar consistently throughout.

- 2nd paragraph of Participants
- 3rd paragraph of Procedure
- 1st paragraph of Analysis
- Results
- Discussion

Without this change in wording, it's a bit confusing to read the study I'm afraid.

 The response document says 'L2 learners' has been changed to 'L2 users'. This doesn't seem consistent through the manuscript. For me, although this is a terminological point, it represents a change in focus. These speakers are the future of Sorbian and are using the language, some of them as prominent community figures by the sound of it. It seems unfair to still call them 'learners', especially at C2 level. This distinction has been discussed for some time in SLA e.g. Cook (1999; https://doi.org/10.2307/3587717) and also applied to language revitalisation contexts e.g. O'Rourke & Ramallo (2013; https://doi.org/10.1017/S0047404513000249) and Ó hIfernáin (2015; https://doi.org/10.1515/ijsl-2014-0031).

 I would consider this an essential change for describing at least the highly advanced C-level speakers.

 I appreciate the clarifications made to the description of the statistics. For the significance testing, I can see that the model summary from the mgcv package has been used for the approximate significance values. It would be good to explain this approach over, for example, model comparison (Sóskuthy 2017; http://eprints.whiterose.ac.uk/113858/). I'm not suggesting that the models should be redone. Indeed this might not be practical or appropriate for the current study. But it would be good to know why this strategy for significance testing was chosen. Perhaps I am not understanding correctly.

 The word list is quite short, which has implications for the interpretation of the results. It is hard to know whether speakers' productions relate to individual words, or relate to production of a system. This could be clearly highlighted in the methods section as well as a short sentence in the Conclusion.

 Ultrasound data were not rotated to the occlusal plane. I accept that not everything can be done, and this can't be changed after data collection! But it should be mentioned in the text.

If applicable, is the statistical analysis and its interpretation appropriate?

Partly

Is the study design appropriate and is the work technically sound?

Yes

Is the work clearly and accurately presented and does it engage with the current literature?

Yes

Are the conclusions drawn adequately supported by the results?

Yes

Are sufficient details of methods and analysis provided to allow replication by others?

Partly

Are all the source data and materials underlying the results available?

Partly

Reviewer Expertise:

Bilingualism, articulatory phonetics, ultrasound and acoustic analysis, sociolinguistics of language revitalisation (Scottish Gaelic in particular).

I confirm that I have read this submission and believe that I have an appropriate level of expertise to confirm that it is of an acceptable scientific standard.

# Reviewer report: Reviewer response for version 2

**DOI:** 10.21956/openreseurope.18664.r38173

**Published:** 2024-03-25

**Authors:** David Bolter

**Affiliations:** 1 Denison University, Granville, Ohio, USA

As I noted in my original review, this article contains an articulatory study of the acquisition of the (voiceless) sibilant system of Lower Sorbian, including /s ʂ ɕ/, by speakers whose L1 or dominant language is German and whose baseline (voiceless) sibilant system includes /s ʃ/. In general, those speakers, whatever level they have on the Common European Framework of Reference, do not produce the unfamiliar /ʂ ɕ/ with distinct tongue profiles, as evidenced by the ultra-sound imaging undertaken by this author. Although most learners at the A, B and C did not produce distinct tongue contours for /ʂ ɕ/, two advanced C-level learners did show evidence of distinct tongue contours for /ʂ ɕ/.

 In assessing the changes to the version of the paper, I think that the author did a sufficient job revising the paper such that I would revise my recommendation to “Accept” with minimal changes necessary.

 I appreciated that the author added more discussion about the fact we are dealing with articulatory data showing no contrast between /ʂ/ and /ɕ/ for the majority of L2 learners. However, it is correct to point out, as Nance & Nagamine do in their review, that this does not necessarily mean that they do not have two categories at the acoustic or perceptual level. I feel, however, that this issue is best addressed with future research projects. It sounds like the author does have audio and video data from the same research project that could be used to further evaluate Sorbian learners’ ability to acquire /ʂ/ and /ɕ/.

 I also feel that a future study could investigate these same individual’s productions in their German. Are they producing German /ʃ/ in the same way as they produce this merged /ʂ ~ ɕ/ category?  

 I also very much appreciated the addition of a German language summary at the top. I’m wondering whether a Sorbian-language could be added and might be even more important to the community.

If applicable, is the statistical analysis and its interpretation appropriate?

I cannot comment. A qualified statistician is required.

Is the study design appropriate and is the work technically sound?

Yes

Is the work clearly and accurately presented and does it engage with the current literature?

Yes

Are the conclusions drawn adequately supported by the results?

Yes

Are sufficient details of methods and analysis provided to allow replication by others?

Yes

Are all the source data and materials underlying the results available?

Yes

Reviewer Expertise:

Phonetics, Phonology, German Dialectology, Historical Linguistics

I confirm that I have read this submission and believe that I have an appropriate level of expertise to confirm that it is of an acceptable scientific standard.

# Reviewer report: Reviewer response for version 1

**DOI:** 10.21956/openreseurope.16097.r34213

**Published:** 2023-08-31

**Authors:** David Bolter

**Affiliations:** 1 Denison University, Granville, Ohio, USA

This article presents an interesting analysis of the degree to which German learners of Lower Sorbian can acquire the contrast between /s ʂ ɕ/ in that language, coming from a system that only contrasts /s ʃ/. The study uses ultra-sound technology and finds that A-Level, B-Level and C-Level learners do not show a contrast in their articulation of (target) /ɕ/ and /ʂ/. Only highly advanced C-level learners demonstrate a clear contrast between (target) /ɕ/ and /ʂ/. However, these highly advanced speakers did not necessarily realize that in a fully target-like manner.

 This is an interesting article that has interesting implications for the kinds of language contact situations where the smaller language is moribund. In my review, I concentrate my energies on questions regarding the type of German that the participants speak (do they contrast /ʃ/ and /ç/?) and the consequences this may have for their acquisition of Sorbian as well as the larger context of language contact situations where the sibilant inventories differ. On the latter point, I wonder specifically whether the authors might be able to offer any predictions on what might happen in some of the cases that I mention.

 My recommendation is that the article be approved pending some revision, with the changes overall being mostly rather minor. I think most of the paper is very clear, but there are some paragraphs that I find confusing specifically in the “Methods” section.

 I further clarify that I have not evaluated the statistical methods undertaken in this article and I encourage further reviewers to address this aspect of the paper.

 Typographic / Clarity suggestions:

 (p. 4-5): The language in the participants section is confusing to me. I’m having a hard time understanding the speakers’ linguistic backgrounds. For example, the authors write “All participants had a first language of German” (p. 5), but then a few sentences later, they discuss the data collected with L1 Sorbian speakers. Perhaps I am not understanding how the authors are L1 speakers, but this seems contradictory to me. Also, what exactly is meant by a “late-acquiring bilingual speaker of Sorbian” (p. 4)?

 (p. 6): “The contours for /ʂ, ɕ/ were more rounded, fronted, and raised than for /s/.”

 In this sentence, my assumption is that “rounded” is referring to rounding or arching of the tongue body, rather than to lip rounding. Is this correct?

 (p. 9) “learners“ is misspelled as “leaners” in the second line under “Discussion”.

 General comments:

 I’m wondering about the system of fricatives used by the participants (and the surrounding area) when speaking (Standard?) German. For example, many speakers in Saxony (cf. AdA: https://www.atlas-alltagssprache.de/runde-2/f25c/) do not contrast between /ʃ/ and /ç/. I suppose Cottbus is a bit outside of this area, but nonetheless establishing their ‘starting point’ in German could be useful in assessing their acquisition. If any speakers are not distinguishing /ʃ/ and /ç/ when speaking Standard German, then I imagine this might affect their ability to learn a contrast between /ʂ/ and /ɕ/.

 Either way, it is sometimes said that Standard German /ʃ/ is labialized [ʃʷ] (Krech et al. 2009: 81-83), perhaps to amplify the contrast with /ç/ (?). If this is so, then a labialized [ʃʷ] might not be all that different from a Slavic [ʂ]. Especially, since some researchers dispute the label of “retroflex” being applied to a language like Polish on the grounds that Polish /ʂ/ has a relatively flat tongue profile (see the debate between Hamann 2004, Żygis et al. 2012 and Ćavar & Lulich 2020).

 I believe that the article could benefit from some mention of this problem, specifically with regards to the hypothesis section. As an ancillary question, I’m wondering whether the data in question could be used to test the goodness of fit of L2 /ɕ/ and /ʂ/ to other L1 sounds (mostly L2 /ɕ/ to L1 /ç/, but a similar problem arises with /ʂ/ and other adjacent sounds in the German inventory).  

 On a very different note, I’m wondering if the authors have any thoughts on other types of language contact situations where the fricative inventories differ. An interesting comparandum (in more ways than one) that the author may wish to consider now or for future work, would be the sibilant systems of Basque in contact with (Peninsular) Spanish (cf. Beristain 2022). Here, the fricative systems in contact are different (Basque /s̺ s̻ ʃ/ vs. Peninsular Spanish /θ s̺/), but the /s̻/ may merge in either direction in different Basque varieties and this may have implications on how Spanish is spoken by those individuals.

 Another language contact situation that could provide an interesting comparison would be the varieties of Sinitic Languages in China. My understanding is that Sinitic languages of the north like Mandarin general contrast /s ʂ ɕ/ , whereas Sinitic languages of the Central and Southern have fewer sibilants. For example, Chen & Gussenhoven (2015) describe Shanghai Chinese as contrasting /s/-series and /ɕ/-series and Hong Kong Cantonese (Zee 1991) has only an /s/-series. My understanding is that L1 speakers of such varieties merge the /s/ and /ʂ/-series when speaking Mandarin.

 All that said, I’m wondering if the author has any predictions regarding how language contact situations with differing sibilant systems such as those referenced in the preceding paragraphs might behave.

If applicable, is the statistical analysis and its interpretation appropriate?

I cannot comment. A qualified statistician is required.

Is the study design appropriate and is the work technically sound?

Yes

Is the work clearly and accurately presented and does it engage with the current literature?

Yes

Are the conclusions drawn adequately supported by the results?

Yes

Are sufficient details of methods and analysis provided to allow replication by others?

Yes

Are all the source data and materials underlying the results available?

Yes

Reviewer Expertise:

Phonetics, Phonology, German Dialectology, Historical Linguistics

I confirm that I have read this submission and believe that I have an appropriate level of expertise to confirm that it is of an acceptable scientific standard, however I have significant reservations, as outlined above.

## References

[1] : Spectral properties of anterior sibilant fricatives in Northern Peninsular Spanish and sibilant-merging and non-merging varieties of Basque. *Journal of the International Phonetic Association* .2022;52(3) : 10.1017/S0025100320000274 421-452 10.1017/S0025100320000274

[2] : Palatalization in coronal consonants of Polish: A three-/four-dimensional ultrasound study. *J Acoust Soc Am* .2020;148(6) : 10.1121/10.0002904 EL447 10.1121/10.0002904 33379916

[3] : Atlas zur deutschen Alltagssprache (AdA).2003;

[4] : Retroflex fricatives in Slavic languages. *Journal of the International Phonetic Association* .2004;34(1) : 10.1017/S0025100304001604 53-67 10.1017/S0025100304001604

[5] : Deutsches Aussprachewörterbuch. *Walter de Gruyter.* .2009;

[6] : Shanghai Chinese. *Journal of the International Phonetic Association* .2015;45(3) : 10.1017/S0025100315000043 321-337 10.1017/S0025100315000043

[7] : Chinese (Hong Kong Cantonese). *Journal of the International Phonetic Association* .1991;21(1) : 10.1017/S0025100300006058 46-48 10.1017/S0025100300006058

[8] : (Non-)retroflex Slavic affricates and their motivation: Evidence from Czech and Polish. *Journal of the International Phonetic Association* .2012;42(3) : 10.1017/S0025100312000205 281-329 10.1017/S0025100312000205

# Response

**Published:** 2024-02-02

**Authors:** Phil Howson

**Affiliations:** Leibniz-Zentrum Allgemeine Sprachwissenschaft, Germany

This article presents an interesting analysis of the degree to which German learners of Lower Sorbian can acquire the contrast between /s ʂ ɕ/ in that language, coming from a system that only contrasts /s ʃ/. The study uses ultra-sound technology and finds that A-Level, B-Level and C-Level learners do not show a contrast in their articulation of (target) /ɕ/ and /ʂ/. Only highly advanced C-level learners demonstrate a clear contrast between (target) /ɕ/ and /ʂ/. However, these highly advanced speakers did not necessarily realize that in a fully target-like manner.

 This is an interesting article that has interesting implications for the kinds of language contact situations where the smaller language is moribund. In my review, I concentrate my energies on questions regarding the type of German that the participants speak (do they contrast /ʃ/ and /ç/?) and the consequences this may have for their acquisition of Sorbian as well as the larger context of language contact situations where the sibilant inventories differ. On the latter point, I wonder specifically whether the authors might be able to offer any predictions on what might happen in some of the cases that I mention.

 My recommendation is that the article be approved pending some revision, with the changes overall being mostly rather minor. I think most of the paper is very clear, but there are some paragraphs that I find confusing specifically in the “Methods” section.

 I further clarify that I have not evaluated the statistical methods undertaken in this article and I encourage further reviewers to address this aspect of the paper. Typographic / Clarity suggestions: (p. 4-5): The language in the participants section is confusing to me. I’m having a hard time understanding the speakers’ linguistic backgrounds. For example, the authors write “All participants had a first language of German” (p. 5), but then a few sentences later, they discuss the data collected with L1 Sorbian speakers. Perhaps I am not understanding how the authors are L1 speakers, but this seems contradictory to me. Also, what exactly is meant by a “late-acquiring bilingual speaker of Sorbian” (p. 4)?

**Response: **I changed L1 to “baseline speakers” to be consistent throughout. The two baseline speakers are also described in more detail: “These participants were chosen for this study because they both had significant input stimuli during the learning process from L1 speakers. Both speakers had input from L1 speaking relatives and additionally the older speaker attended the Sorbian school at a time when L1 Sorbian speaking teachers were active. Additionally, at the time of recording this data, few L1 speakers remain, and the advanced age of potential participants (above 80 years of age) makes ultrasound data especially difficult to record and interpret.”

 (p. 6): “The contours for /ʂ, ɕ/ were more rounded, fronted, and raised than for /s/.” In this sentence, my assumption is that “rounded” is referring to rounding or arching of the tongue body, rather than to lip rounding. Is this correct?

**Response:** Now reads: “The contours for /ʂ, ɕ/ had more rounded tongue shape, with more fronting, and more posterior tongue body raising than for /s/.”

 (p. 9) “learners“ is misspelled as “leaners” in the second line under “Discussion”.

**Response: **Corrected.

 General comments: I’m wondering about the system of fricatives used by the participants (and the surrounding area) when speaking (Standard?) German. For example, many speakers in Saxony (cf. AdA: https://www.atlas-alltagssprache.de/runde-2/f25c/) do not contrast between /ʃ/ and /ç/. I suppose Cottbus is a bit outside of this area, but nonetheless establishing their ‘starting point’ in German could be useful in assessing their acquisition. If any speakers are not distinguishing /ʃ/ and /ç/ when speaking Standard German, then I imagine this might affect their ability to learn a contrast between /ʂ/ and /ɕ/. Either way, it is sometimes said that Standard German /ʃ/ is labialized [ʃʷ] (Krech et al. 2009: 81-83), perhaps to amplify the contrast with /ç/ (?). If this is so, then a labialized [ʃʷ] might not be all that different from a Slavic [ʂ]. Especially, since some researchers dispute the label of “retroflex” being applied to a language like Polish on the grounds that Polish /ʂ/ has a relatively flat tongue profile (see the debate between Hamann 2004, Żygis et al. 2012 and Ćavar & Lulich 2020).

**Response:** This is difficult to say with the current data set. What can be ascertained from the data provided here is that there is tremendous variation in the way individual speakers produce /ʂ, ɕ/ and that overwhelmingly the tongue contours overlap in a way as to suggest no significant differences in the way they are articulated.

 I believe that the article could benefit from some mention of this problem, specifically with regards to the hypothesis section. As an ancillary question, I’m wondering whether the data in question could be used to test the goodness of fit of L2 /ɕ/ and /ʂ/ to other L1 sounds (mostly L2 /ɕ/ to L1 /ç/, but a similar problem arises with /ʂ/ and other adjacent sounds in the German inventory). 

**Response:** With the current dataset, it’s not really possible to do this type of comparison. It is an interesting suggestion and examinations of German L1 learners of a language with the three-way sibilant contrast would benefit strongly from incorporating these data.

 On a very different note, I’m wondering if the authors have any thoughts on other types of language contact situations where the fricative inventories differ. An interesting comparandum (in more ways than one) that the author may wish to consider now or for future work, would be the sibilant systems of Basque in contact with (Peninsular) Spanish (cf. Beristain 2022). Here, the fricative systems in contact are different (Basque /s̺ s̻ ʃ/ vs. Peninsular Spanish /θ s̺/), but the /s̻/ may merge in either direction in different Basque varieties and this may have implications on how Spanish is spoken by those individuals. Another language contact situation that could provide an interesting comparison would be the varieties of Sinitic Languages in China. My understanding is that Sinitic languages of the north like Mandarin general contrast /s ʂ ɕ/ , whereas Sinitic languages of the Central and Southern have fewer sibilants. For example, Chen & Gussenhoven (2015) describe Shanghai Chinese as contrasting /s/-series and /ɕ/-series and Hong Kong Cantonese (Zee 1991) has only an /s/-series. My understanding is that L1 speakers of such varieties merge the /s/ and /ʂ/-series when speaking Mandarin. All that said, I’m wondering if the author has any predictions regarding how language contact situations with differing sibilant systems such as those referenced in the preceding paragraphs might behave.

**Response: **I think language contact situations can differ significantly based on the communities. Certainly, there are theoretical considerations along with those, such that we may assume or observe tendencies towards certain patterns of acquisition and assimilation. In keeping with the theoretical claims in this paper, the most likely assimilatory patterns are likely those that adhere to the assimilation of segments with the greatest acoustic-perceptual overlap.

# Reviewer report: Reviewer response for version 1

**DOI:** 10.21956/openreseurope.16097.r33691

**Published:** 2023-08-25

**Authors:** Claire Nance, Takayuki Nagamine

**Affiliations:** 1 Linguistics and English Language, Lancaster University, Lancaster, England, UK

**Summary**

 This is an articulatory study which considers L1 German speakers’ production of fricatives in L2 Lower Sorbian speech. The study analyses production of Lower Sorbian /s ʂ ɕ/, hypothesising that L1 German - L2 Lower Sorbian speakers would produce Lower Sorbian /ʂ ɕ/ similarly to German /ʃ/ due to their acoustic-perceptual similarity. Effect of L2 proficiency is also considered.

 The author analyses ultrasound tongue imaging data obtained from Lower Sorbian speakers at different proficiency levels. Tongue splines from Sorbian fricative midpoints were extracted at the acoustically defined fricative midpoint and analysed with polar GAMMs.

 L1 speakers showed three-way contrasts in Lower Sorbian /s ʂ ɕ/ whereas the majority of L2 speakers exhibited two-way contrasts between /s/ and /ʂ ɕ/, arguably due to influence of L1 German. Only advanced L2 users of Lower Sorbian produced the three-way fricative contrast.

**Strengths of this study**

 It is wonderful to see more articulatory work on Sorbian! It’s so important to have this kind of language documentation, as well as a detailed investigation of speech production in a language revitalisation setting.

 We haven’t previously seen analysis of language revitalisation users at different proficiency levels, so this is an important contribution of the work.

 There is very little articulatory work carried out with bilinguals/L2 users, so this work is a significant addition to the field.

 We suggest some revisions to the final version of the paper. This mainly concerns some rephrasing of the framing, more details on aspects of the methods and analysis, and greater acknowledgement of what can and can’t be concluded from this dataset.

**Major points**

 Sociolinguistically, this is a context of extreme language endangerment and some revitalisation. It is to be expected that speakers using the language as an L2 won’t sound exactly the same as older people who acquired it in a completely different social context. Suggest some rephrasing and acknowledgement of this throughout. For example, removing references to speakers not producing sounds ‘properly’ etc. Replace with them sounding ‘different’ to Sorbian acquired two generations ago in an L1 setting.

 We appreciate that sociolinguistics is not the primary framing of the work here, but if you wanted to consider this aspect in more detail, you could investigate work conducted in the new speaker framework. It seems that ‘new speakers’ would be a very appropriate way to describe these Sorbian L2 users. Some papers we found helpful:

 O’Rourke & Ramallo. (2011). https://doi.org/10.1075/lplp.35.2.03oro

 Jaffe. (2015). https://doi.org/10.1515/ijsl-2014-0030

**Hypothesis:**

 This section gives predictions for the study based on the PAM-L2 and SLM(-r). However, the current study is all about articulation with no examination of the acoustic data. Some thinking needs to go into this about the targets that speakers are aiming for. Are they aiming for an acoustic target or an articulatory target in acquiring Sorbian?

 Our understanding is that while the PAM-L2 has a prediction about articulation (that speakers directly perceive gestures and acquire these), the SLM-r is less specific about articulation. Clarify this distinction somewhere in the lit review as it is important for the hypothesis here.

 In the hypothesis paragraph, you mention quite a lot about formant transitions and spectral characteristics of the fricatives. This can’t be tested in the current design, which is purely articulatory. We suggest rethinking about how this material fits into the articulatory message of this paper, and the links between acoustics and articulation.

 This is important for the rest of the study. For example, on p9 you refer to speakers not acquiring the contrast. The data here refer to midsagittal tongue splines at fricative midpoint only. It is possible that speakers are making an acoustic contrast in some other way. Unlikely perhaps, but some thought needs to go into what we can and can’t ascertain from these data.

**Discussion:**

 There is an argument here and in the Results that Sorbian speakers are substituting German /ʃ/ for two of the Sorbian fricatives. This seems impossible to know from the current data, since German /ʃ/ isn’t analysed. It is possible that Sorbian learners are doing something that is different from L1 Sorbian and also different from German. See Moore et al. (2018) for a similar example where Japanese L1 English L2 speakers produce a sound which is not like L1-English /l/ and /ɹ/, but is also different from their Japanese [ɾ].

 Moore et al. (2018). https://doi.org/10.1250/ast.39.75

**Open Science:**

 We are grateful to the author for providing the datasheets used. In order for future researchers to fully replicate this study and understand all the statistics carried out, it would be very helpful to have the code used as well.

**Minor points**

**Abstract:**

 This reads more like the first page of the Introduction. It would be helpful to make this more of an ‘advert’ for the study. Include more of the findings here. A major contribution of the work, and a major feature of the study design, is comparing the different proficiency levels for speakers. This should be mentioned in the Abstract.

**Plain language summary:**

 This is still quite technical for non-linguists to read, for example references to IPA terminology. Suggest some rephrasing such that this summary would be useful to Sorbian community members (e.g. give example words for IPA). Would it be possible to translate into German and/or Sorbian as well for maximum readability by Sorbian users?

**Introduction:**

 2 ^nd^ paragraph: Is it possible to quantify how uncommon a three-way sibilant contrast is?

 For example, look through Maddieson (1984) *Patterns of sounds*, or the P-database: https://pbase.phon.chass.ncsu.edu

 A lot in this analysis hangs on /ʂ/ and /ɕ/ being more acoustically/perceptually similar to /ʃ/ than /s/. Is there any way you can demonstrate this?

**Second language acquisition:**

 The material in this section comes a bit abruptly after the Introduction. Could it be linked in a little more?

**PAM-L2:**

 P3, final paragraph, column 2: Suggest rephrasing ‘poverty of stimulus’. We’re talking about a highly endangered language where people are doing their very best to revitalise and transmit the language however they can. A classroom will be different to a home setting, but ‘poverty of stimulus’ sounds very negative, perhaps unnecessarily so.

 Similarly, the reference to teachers ‘not properly producing’ target segments. Maybe rephrase to acknowledge that L2 users will speak differently to L1 users, but this is what we would expect anyway.

 Could the discussion about lexical frequency and word learning vs. category learning (Tyler 2019) be linked into the current study? This material seems perhaps less relevant for a word list task such as the current work. See also comments further down about the stimuli.

**Speech Learning Model:**

 Second paragraph tells us that the target population are late-acquiring bilinguals. Mention this earlier.

 It would be good to define what is meant by ‘late-acquiring bilingual’.

**Methods:**

**Study design:**

 No need to mention the quiet room as this is repeated later on.

**Participants:**

 We were a bit confused about the baseline participants. Are they the L1 speakers? You mentioned earlier that most L1 speakers are elderly.

 As one of them acquired Sorbian from age 5 (if we understand correctly), could you give a little more information about their language acquisition trajectory? How were they selected as representative of L1 Sorbian?

**Figure 1:**

 Could you give a bit more information about what is shown in this figure? Are the arrows relevant to a particular bit of the waveform? What do the spectrograms show here which informs the reader beyond IPA symbols and arrows? Suggest changing to a schematised IPA diagram instead unless the spectrograms illustrate something specific.

**Participants p5:**

 Could you specify the age and gender of participants in the main text instead of the supplementary materials, as this seems like key information. Explain a little bit more about language learning trajectories and how some participants have spent a long time at A-level compared to others who have reached B-level comparatively faster. In a context of language revitalisation learning, this isn’t entirely surprising, but might be less evident to readers coming from a majority-language teaching background.

 How was the level of the learners assessed? Is this through a standardised test or teacher perceptions?

 Make it clear that these scores refer to CEFR scales (we assume), otherwise this can be a bit confusing. For example, as a British reader, it is hard not to read ‘A-Level’ as referring to the standard tests taken by 18-year-old school leavers in most of the UK. These are referred to as ‘A-Levels’.

 How was the level of the advanced C2 speakers ascertained?

 Is it still appropriate to refer to them as ‘learners’ if they are so advanced? Suggest replacing with ‘L2 users’ or ‘L2 speakers’.

 ‘one of which has achieved a near-native level of fluency’ – how was this ascertained?

 Suggest replacing with ‘L1-like level of fluency’.

**Procedure:**

 Second paragraph at the bottom of p5: ‘Data for the bilingual speakers’ – aren’t all of them bilingual?

**Stimuli:**

 This is quite a small list for a study to make claims about category learning. As there is only one word per context, it is not possible to disentangle category learning, and learning of individual words. Some of the words are presumably more frequent than others, which will likely affect acquisition for the less advanced speakers. This needs to be acknowledged in the study text.

**Analysis:**

 It would be helpful to have a little more information about the ultrasound data here:

 Was any manual correction carried out for the tongue splines obtained from AAA?

 Were the data rotated to the occlusal plane?

 Which version of AAA was used for the analysis?

 Could you give a bit more information about how significance testing was carried out? Via model comparison?

**Results:**

**A-Level learners:**

 ‘A-Level learners share one phoneme’ – rephrase. They share one tongue shape. We don’t know about the perceptual categories needed for phoneme-hood.

 Final paragraph of this section: is the ‘significant variation in their articulation of /ʃ/’ a typo?

**B-Level learners:**

 ‘have not acquired the three-way contrast’ – make it clear that this refers to articulation, as we don’t know about their acoustics/perception from this study.

 First paragraph on p9: ‘B-level learners continue to use German /ʃ/’. It’s not possible to draw this conclusion from these data as they don’t include analysis of German /ʃ/.

 /ʃ/ often involves significant lip rounding. This study doesn’t consider lip rounding analysis. This should be taken into account when drawing conclusions about likely German influence.

**Highly advanced C-Level learners:**

 It seems there is some inconsistency in labelling these participants between the main text, and the supplementary materials. Consider using ‘C04’ and ‘C05’ throughout instead of ‘C201’ and ‘C202’.

**Tongue spline figures:**

 Consider changing the red/orange colour scheme to maximise contrast for readers.

**Discussion:**

 It is suggested here that L2 Sorbian learners experience ‘L1 interference in the acoustic-perceptual space’. But at the same time, the text says that Sorbian teachers might not produce this contrast themselves. So, it is possible that learners are doing a really amazing job of repeating exactly what their teachers produce and aren’t experiencing L1 influence directly themselves at all. Could you explain how these things can be disentangled, or what is the most likely interpretation of the data?

 ‘contrasts with difficult to perceive differences require specific training to acquire’. Could you give some evidence from the L2 pronunciation literature to back this up? This isn’t my exact area of specialism, but my impression is that L2 pronunciation training is extremely difficult and might not be ‘successful’ in creating L1-like pronunciations.

 At the same time, thought needs to be given to what is a realistic target for L2 users and language revitalisation speakers. In applied circles, it is now usually considered more appropriate to aim for intelligible and comprehensible L2 speech, rather than an L1 target for pronunciation.

 You conclude that intervention and training is needed here and in the plain language summary. But the advanced C-Level speakers did produce this contrast without having had special training. It could therefore be argued that more language exposure and use is required instead.

If applicable, is the statistical analysis and its interpretation appropriate?

Partly

Is the study design appropriate and is the work technically sound?

Yes

Is the work clearly and accurately presented and does it engage with the current literature?

Yes

Are the conclusions drawn adequately supported by the results?

Yes

Are sufficient details of methods and analysis provided to allow replication by others?

Partly

Are all the source data and materials underlying the results available?

Partly

Reviewer Expertise:

Claire Nance and Takayuki Nagamine reviewed this article. We work in bilingualism and articulatory phonetics. We use ultrasound and acoustic analysis as research methods. Claire has expertise in sociolinguistics of language revitalisation (Scottish Gaelic in particular). Takayuki has expertise in language teaching and L2 pronunciation (Japanese-English bilinguals and English teaching).

We confirm that we have read this submission and believe that we have an appropriate level of expertise to confirm that it is of an acceptable scientific standard, however we have significant reservations, as outlined above.

## References

[1] : The native-non-native dichotomy in minority language contexts. *Language Problems and Language Planning* .2011;35(2) : 10.1075/lplp.35.2.03oro 139-159 10.1075/lplp.35.2.03oro

[2] : Defining the new speaker: theoretical perspectives and learner trajectories. *International Journal of the Sociology of Language* .2015;2015(231) : 10.1515/ijsl-2014-0030 21-44 10.1515/ijsl-2014-0030

[3] : Articulation strategies for English liquids used by Japanese speakers. *Acoustical Science and Technology* .2018;39(2) : 10.1250/ast.39.75 75-83 10.1250/ast.39.75

[4] : Patterns of sounds. *Cambridge University Press* .1984;

# Response

**Published:** 2024-02-02

**Authors:** Phil Howson

**Affiliations:** Leibniz-Zentrum Allgemeine Sprachwissenschaft, Germany

This is an articulatory study which considers L1 German speakers’ production of fricatives in L2 Lower Sorbian speech. The study analyses production of Lower Sorbian /s ʂ ɕ/, hypothesising that L1 German - L2 Lower Sorbian speakers would produce Lower Sorbian /ʂ ɕ/ similarly to German /ʃ/ due to their acoustic-perceptual similarity. Effect of L2 proficiency is also considered. The author analyses ultrasound tongue imaging data obtained from Lower Sorbian speakers at different proficiency levels. Tongue splines from Sorbian fricative midpoints were extracted at the acoustically defined fricative midpoint and analysed with polar GAMMs. L1 speakers showed three-way contrasts in Lower Sorbian /s ʂ ɕ/ whereas the majority of L2 speakers exhibited two-way contrasts between /s/ and /ʂ ɕ/, arguably due to influence of L1 German. Only advanced L2 users of Lower Sorbian produced the three-way fricative contrast. Strengths of this study It is wonderful to see more articulatory work on Sorbian! It’s so important to have this kind of language documentation, as well as a detailed investigation of speech production in a language revitalisation setting. We haven’t previously seen analysis of language revitalisation users at different proficiency levels, so this is an important contribution of the work. There is very little articulatory work carried out with bilinguals/L2 users, so this work is a significant addition to the field. We suggest some revisions to the final version of the paper. This mainly concerns some rephrasing of the framing, more details on aspects of the methods and analysis, and greater acknowledgement of what can and can’t be concluded from this dataset. *Major points* Sociolinguistically, this is a context of extreme language endangerment and some revitalisation. It is to be expected that speakers using the language as an L2 won’t sound exactly the same as older people who acquired it in a completely different social context. Suggest some rephrasing and acknowledgement of this throughout. For example, removing references to speakers not producing sounds ‘properly’ etc. Replace with them sounding ‘different’ to Sorbian acquired two generations ago in an L1 setting.

**Response:** Thank you for this, I think it is an important change to make. I have changed the language throughout.

 We appreciate that sociolinguistics is not the primary framing of the work here, but if you wanted to consider this aspect in more detail, you could investigate work conducted in the new speaker framework. It seems that ‘new speakers’ would be a very appropriate way to describe these Sorbian L2 users. Some papers we found helpful: O’Rourke & Ramallo. (2011). https://doi.org/10.1075/lplp.35.2.03oro Jaffe. (2015). https://doi.org/10.1515/ijsl-2014-0030   Hypothesis: This section gives predictions for the study based on the PAM-L2 and SLM(-r). However, the current study is all about articulation with no examination of the acoustic data. Some thinking needs to go into this about the targets that speakers are aiming for. Are they aiming for an acoustic target or an articulatory target in acquiring Sorbian?

**Response:** I have added the following text: “In terms of the PAM-L2 (Best & Tyler, 2007), the assumption is that learners are perceiving articulatory gestures and vocal tract changes, not more abstract acoustic characteristics. The implication of this is that as learners become more advanced, they become better at retrieving the articulatory movements necessary to produce a contrast. The expectation is that gradual improvement in the articulation of L2 segments should occur.”

 Our understanding is that while the PAM-L2 has a prediction about articulation (that speakers directly perceive gestures and acquire these), the SLM-r is less specific about articulation. Clarify this distinction somewhere in the lit review as it is important for the hypothesis here.

**Response:** I have added the following text: “The PAM-L2 is a direct realist model, which assumes that perception is related to the perception of distal articulatory events (i.e., changes in vocal tract configurations), not specific acoustic patterns.” and: “Additionally, the PAM-L2 posits that learners attenuating to gestural movements in the vocal tract, while the SLM-r suggests that learners pay attention to acoustic differences in the input signal directly. Thus, under the view of the SLM-r, articulation is a matter of better navigation of what vocal tract shapes produce the target acoustic outputs.”

 In the hypothesis paragraph, you mention quite a lot about formant transitions and spectral characteristics of the fricatives. This can’t be tested in the current design, which is purely articulatory. We suggest rethinking about how this material fits into the articulatory message of this paper, and the links between acoustics and articulation.

**Response:** I have added the following text: “In terms of the PAM-L2 (Best & Tyler, 2007), the assumption is that learners are perceiving articulatory gestures and vocal tract changes, not more abstract acoustic characteristics. The implication of this is that as learners become more advanced, they become better at retrieving the articulatory movements necessary to produce a contrast. The expectation is that gradual improvement in the articulation of L2 segments should occur. In terms of the SLM-r (Flege & Bohn, 2021), there is a similar expectation. As learners’ acoustic-perceptual representation improves, so too should articulation.”

 This is important for the rest of the study. For example, on p9 you refer to speakers not acquiring the contrast. The data here refer to midsagittal tongue splines at fricative midpoint only. It is possible that speakers are making an acoustic contrast in some other way. Unlikely perhaps, but some thought needs to go into what we can and can’t ascertain from these data. Discussion: There is an argument here and in the Results that Sorbian speakers are substituting German /ʃ/ for two of the Sorbian fricatives. This seems impossible to know from the current data, since German /ʃ/ isn’t analysed. It is possible that Sorbian learners are doing something that is different from L1 Sorbian and also different from German. See Moore et al. (2018) for a similar example where Japanese L1 English L2 speakers produce a sound which is not like L1-English /l/ and /ɹ/, but is also different from their Japanese [ɾ]. Moore et al. (2018). https://doi.org/10.1250/ast.39.75   Open Science: We are grateful to the author for providing the datasheets used. In order for future researchers to fully replicate this study and understand all the statistics carried out, it would be very helpful to have the code used as well.

**Response:** The code is included: “ (1) r ~ Segment * Environment + s(Theta, bs = “cr”, k = 25) + s(Theta, by = Segment, bs = “cr”, k = 25) + s(Theta, by = Segment : Environment, bs = “cr”, k = 25) + s(Theta, by = Segment : Speaker, bs = “fs”, k = 25, m = 1)”      “(2) r ~ Segment * Environment + s(Theta, bs = “cr”, k = 20) + s(Theta, by = Segment, bs = “cr”, k = 20) + s(Theta, by = Segment : Environment, bs = “cr”, k = 20) + s(Theta, by = Rep, bs = “fs”, k = 20, m = 1)” These models are clearly written such that they are easily producible with datasheet given.

 Minor points Abstract: This reads more like the first page of the Introduction. It would be helpful to make this more of an ‘advert’ for the study. Include more of the findings here. A major contribution of the work, and a major feature of the study design, is comparing the different proficiency levels for speakers. This should be mentioned in the Abstract.

**Response:** I have added the following: “The ultrasound data revealed that learners in the contemporary context do not produce a distinction between /ʂ, ɕ/ and only learners at an advanced level who had significant exposure to L1 speakers have acquired a three-way sibilant distinction.”

 Plain language summary: This is still quite technical for non-linguists to read, for example references to IPA terminology. Suggest some rephrasing such that this summary would be useful to Sorbian community members (e.g. give example words for IPA). Would it be possible to translate into German and/or Sorbian as well for maximum readability by Sorbian users?

**Response:** I have added the following example: “Both of these segments are perceptually similar to the German sibilant fricative common represented with sch (e.g., Schlange snake).” I have also changed the following text to read: “The results implicate that in a language revitalization context where few L1 speakers are available, the input that learners receive should be augmented with pronunciation and perceptual resources to assist in acquisition. Specific recommendations are provided.” A German translation is also provided: “Auf Deutsch Der Zweitspracherwerb setzt voraus, dass die Sprachlernenden eine Reihe neuer Sprachsegmente erwerben. Junge Sorben, die Niedersorbisch als Zweitsprache lernen, müssen zwei neue Zischlaute (hochfrequente, laute Segmente wie /s/) erwerben. Diese beiden Segmente ähneln in der Wahrnehmung dem deutschen Zischlaut, der häufig mit sch dargestellt wird (z.B. Schlange). In dieser Studie wird der Erwerb der niedersorbischen Zischlautkontraste mittels Ultraschalltechnologie untersucht. Ultraschall zeichnet Videos von Zungenkonturen mit einer hohen Bildrate auf, so dass eine statistische Analyse der Zungenformen durchgeführt werden kann. In diesem Projekt untersuche ich die Zungenkonturen niedersorbischer Lerner auf der Anfänger-, Mittelstufen- und Fortgeschrittenenstufe, um zu beobachten, wie Zungenformen für Zischlaute erworben werden. Die Ergebnisse deuten darauf hin, dass in einem Sprachwiederbelebungskontext, in dem nur wenige L1-Sprecher zur Verfügung stehen, der Input, den die Lernenden erhalten, mit Aussprache- und Wahrnehmungsressourcen ergänzt werden sollte, um den Erwerb zu unterstützen. Es werden spezifische Empfehlungen gegeben.”

 Introduction: 2nd paragraph: Is it possible to quantify how uncommon a three-way sibilant contrast is? For example, look through Maddieson (1984) Patterns of sounds, or the P-database: https://pbase.phon.chass.ncsu.edu

**Response:** From what I can derive from Maddieson (1984), specific numbers on the three-way sibilant contrast are not provided. Numbers for languages with one or more fricative are provided, but reference to which segment that is are not. This is more complex when voiced and voiceless pairs are considered 2 segments and non-sibilant segments are included. Thus, minimally, we would expect that any of the numbers for 6+ fricatives could apply, but the numbers then vary. I could estimate, that in all likelihood, of the languages examined by Maddieson (1984), less than 6% have a three-way sibilant contrast. That being said, this would be an estimation based on the available data.

 A lot in this analysis hangs on /ʂ/ and /ɕ/ being more acoustically/perceptually similar to /ʃ/ than /s/. Is there any way you can demonstrate this?

**Response:** I have added the following to page ?: “The acoustics between the two segments /ʂ, ɕ/ resemble each other across in COG and skewness, having both a lower COG and higher skewness than /s/. Both values also significantly overlapped with each other for /ʂ, ɕ/. The feature in Lower Sorbian that was found to most strongly distinguish /ʂ, ɕ/ from each other was a much higher transitional F2 into the following vowel for /ɕ/ compared to /ʂ/ (Howson, 2015). The lower COG values observed in Lower Sorbian, tend to match cross-linguistic COG associated with /ʃ/ (Żygis, 2010) and COG and skewness measures associated with German /ʃ/ (Weirich & Simpson, 2015).”

 Second language acquisition: The material in this section comes a bit abruptly after the Introduction. Could it be linked in a little more?

**Response:** I have added the following: “Many theories of language acquisition, such as the PAM-L2 (Best & Tyler, 2007) and the SLM-r (Flege & Bohn, 2021), have postulated how different aspects of acoustic-perceptual similarities with L1 segments impacts L2 acquisition. Contrasts such as the three-way contrast, under these theories are the most difficult to acquire due to the acoustic similarities between the segments. This makes Lower Sorbian an excellent language to examine foreign language acquisition of sibilant fricatives.”

 PAM-L2: P3, final paragraph, column 2: Suggest rephrasing ‘poverty of stimulus’. We’re talking about a highly endangered language where people are doing their very best to revitalise and transmit the language however they can. A classroom will be different to a home setting, but ‘poverty of stimulus’ sounds very negative, perhaps unnecessarily so.

**Response:** I have changed this to: “The reason for this is because of a reduced access to consistent stimuli and the phonetic contrasts that distinguish them.”

 Similarly, the reference to teachers ‘not properly producing’ target segments. Maybe rephrase to acknowledge that L2 users will speak differently to L1 users, but this is what we would expect anyway.

**Response:** I have changed this to read the following: “Many second language classrooms are also taught by second language speakers, who may or may not consistently produce the language relevant contrasts, and likely produce contrasts differently than the older generation of L1 speakers.”

 Could the discussion about lexical frequency and word learning vs. category learning (Tyler 2019) be linked into the current study? This material seems perhaps less relevant for a word list task such as the current work. See also comments further down about the stimuli.   Speech Learning Model: Second paragraph tells us that the target population are late-acquiring bilinguals. Mention this earlier. It would be good to define what is meant by ‘late-acquiring bilingual’.

**Response:** I have added the following: “The SLM posits that for late acquiring bilinguals (i.e., someone who acquired two languages as a child, but the second language was acquired later than the first)”

 Methods: Study design: No need to mention the quiet room as this is repeated later on.

**Response: **Removed.

 Participants: We were a bit confused about the baseline participants. Are they the L1 speakers? You mentioned earlier that most L1 speakers are elderly. As one of them acquired Sorbian from age 5 (if we understand correctly), could you give a little more information about their language acquisition trajectory? How were they selected as representative of L1 Sorbian?

**Response:** Both speakers acquired the languages in a bilingual context and as a minority language. That is, German was the primarily language of the household, but they both had access to first language speakers through family and friends and through earlier integration into the Sorbian school system when L1 teachers were still available. There are two reasons L1 speakers were not included. The first, there are very few and they are very old. This means that most L1 speakers have no real interest in participating in any linguistic (or other) studies on Lower Sorbian. The second reason is that for speakers at such an advanced age, it is extremely difficult to obtain a quality recording. This is one of the draw backs of ultrasound in general and is something that has to be considered. I have added the following text: “These participants were chosen for this study because they both had significant input stimuli during the learning process from L1 speakers. Both speakers had input from L1 speaking relatives and additionally the older speaker attended the Sorbian school at a time when L1 Sorbian speaking teachers were active. Additionally, at the time of recording this data, few L1 speakers remain, and the advanced age of potential participants (above 80 years of age) makes ultrasound data especially difficult to record and interpret.”

 Figure 1: Could you give a bit more information about what is shown in this figure? Are the arrows relevant to a particular bit of the waveform? What do the spectrograms show here which informs the reader beyond IPA symbols and arrows? Suggest changing to a schematised IPA diagram instead unless the spectrograms illustrate something specific.

**Response:** The figure simply illustrated some observable similarities in center of gravity, however, above I now provide citations and values that compare these segments (see above for specific text added) and it is perhaps no longer necessary. I have removed this figure and renumbered the other figures accordingly.

 Participants p5: Could you specify the age and gender of participants in the main text instead of the supplementary materials, as this seems like key information. Explain a little bit more about language learning trajectories and how some participants have spent a long time at A-level compared to others who have reached B-level comparatively faster. In a context of language revitalisation learning, this isn’t entirely surprising, but might be less evident to readers coming from a majority-language teaching background.  

 How was the level of the learners assessed? Is this through a standardised test or teacher perceptions?

**Response:** There are no real standardized tests for assessing Sorbian abilities. It had only to do with their level within the Sorbian school system. I have added the following text: “Participants were recruited for all three skill levels, A-, B-, and C-level learners based on a scaling system like the CEFR. Their skill level at the time was based on class they attended for Sorbian language at gymnazium Chóśebuz at the time of recording.”

 Make it clear that these scores refer to CEFR scales (we assume), otherwise this can be a bit confusing. For example, as a British reader, it is hard not to read ‘A-Level’ as referring to the standard tests taken by 18-year-old school leavers in most of the UK. These are referred to as ‘A-Levels’.

**Response:** See above for additional text.

 How was the level of the advanced C2 speakers ascertained?

**Response:** Both speakers are well known in the Sorbian community (I prefer not to provide too many identifying details to maintain anonymity). But I can say that both are well known speakers.

 Is it still appropriate to refer to them as ‘learners’ if they are so advanced? Suggest replacing with ‘L2 users’ or ‘L2 speakers’.

**Response:** Changed.

 ‘one of which has achieved a near-native level of fluency’ – how was this ascertained?

**Response:** Again, this is generally accepted within the community, and they are well known for this. However, perhaps this information is too much as people who know them might easily figure out who they are. I have removed it.

 Suggest replacing with ‘L1-like level of fluency’.

**Response: **Removed the reference.

 Procedure: Second paragraph at the bottom of p5: ‘Data for the bilingual speakers’ – aren’t all of them bilingual?

**Response:** Changed this to “baseline speakers.”

 Stimuli: This is quite a small list for a study to make claims about category learning. As there is only one word per context, it is not possible to disentangle category learning, and learning of individual words. Some of the words are presumably more frequent than others, which will likely affect acquisition for the less advanced speakers. This needs to be acknowledged in the study text.

**Response: **The following has been added to the conclusions: “However, it should be noted that one limitation of the study is the relatively small wordlist which makes it more difficult to assess category formation.”

 Analysis: It would be helpful to have a little more information about the ultrasound data here: Was any manual correction carried out for the tongue splines obtained from AAA?

**Response:** Tongue traces were all manual. I have added that in the text. This was largely done before I had DeepLabCut installed and operational.

 Were the data rotated to the occlusal plane?

**Response:** No.

 Which version of AAA was used for the analysis?

**Response:** R was used for the analysis and is now included in the citations. Version data for AAA also included in the text.

 Could you give a bit more information about how significance testing was carried out? Via model comparison?

**Response:** The following text was included: “GAMMs were performed using the mgcv package (Wood, 2011), which also provides summary statistics.”

 Results: A-Level learners: ‘A-Level learners share one phoneme’ – rephrase. They share one tongue shape. We don’t know about the perceptual categories needed for phoneme-hood.

**Response:** Changed.

 Final paragraph of this section: is the ‘significant variation in their articulation of /ʃ/’ a typo?

**Response:** Changed to read /ʂ, ɕ/.

 B-Level learners: ‘have not acquired the three-way contrast’ – make it clear that this refers to articulation, as we don’t know about their acoustics/perception from this study.

**Response:** Now reads: “This suggests that like the A-level learners, the B-level learners also have not acquired the three-way contrast between /s, ʂ, ɕ/ with respect to their articulation.”

 First paragraph on p9: ‘B-level learners continue to use German /ʃ/’. It’s not possible to draw this conclusion from these data as they don’t include analysis of German /ʃ/.

**Response:** Change to read “the same segment.”

 /ʃ/ often involves significant lip rounding. This study doesn’t consider lip rounding analysis. This should be taken into account when drawing conclusions about likely German influence.

**Response:** Thank you for this comment.

 Highly advanced C-Level learners: It seems there is some inconsistency in labelling these participants between the main text, and the supplementary materials. Consider using ‘C04’ and ‘C05’ throughout instead of ‘C201’ and ‘C202’.

**Response:** Changed.

 Tongue spline figures: Consider changing the red/orange colour scheme to maximise contrast for readers.

**Response:** Perhaps the only issue with this is that I was required to register the OSF database prior to receiving these comments. As a result, it is no longer possible to change them and there would then be inconsistencies between plot colors in the text and in the supplementary materials.

 Discussion: It is suggested here that L2 Sorbian learners experience ‘L1 interference in the acoustic-perceptual space’. But at the same time, the text says that Sorbian teachers might not produce this contrast themselves. So, it is possible that learners are doing a really amazing job of repeating exactly what their teachers produce and aren’t experiencing L1 influence directly themselves at all. Could you explain how these things can be disentangled, or what is the most likely interpretation of the data?

**Response: **In reality, it is perhaps more likely that the instructors themselves do not reliably produce the three-way sibilant contrasts and this is one of the starkest contrasts between C04/C05 and the rest of the learners. They had explicit instruction from L1 speakers who consistently produced those contrasts, while the contemporary learners likely do not receive that input in any consistent way, if at all. I have added the following text: “One caveat to note is that the current L2 instructors do not have the level of fluency as the L1 instructors that the two advanced speakers (C04 and C05) had access to. As such, it is difficult to interpret how much input for the three-way sibilant contrast (if any) learners received. It is clear from discussions with the learners that pronunciation lessons are not a regular part of the curriculum. It remains very possible the lack of acquisition of the three-way contrast is predominantly due to lack of the three-way contrast in the input for learners.”

 ‘contrasts with difficult to perceive differences require specific training to acquire’. Could you give some evidence from the L2 pronunciation literature to back this up? This isn’t my exact area of specialism, but my impression is that L2 pronunciation training is extremely difficult and might not be ‘successful’ in creating L1-like pronunciations.

**Response:** This specific line was removed. However, L1-like pronunciation may be relatively unachievable for a second language learner for a variety of reasons (at least “perfect” L1-pronunciation). But for example, even children who learn multiple languages from birth, do not have the same pronunciation as someone who learned only 1 language as a child. It may perhaps not be the best measure of learning then to say that they do or do not speak like a monolingual or the same as someone who learned only 1 language as a child. Rather, in some sense it is better to examine to what degree and how contrasts are made and learned by L2 learners.

 At the same time, thought needs to be given to what is a realistic target for L2 users and language revitalisation speakers. In applied circles, it is now usually considered more appropriate to aim for intelligible and comprehensible L2 speech, rather than an L1 target for pronunciation.

**Response:** I think this depends on the specific context and goals/aims of the group in question. From personal work, I can say it is very important to many indigenous communities that they maintain pronunciation similar/the same as it use to be spoken. I would not say this is an unachievable goal, but if it is the goal, then certainly specific measures to achieve that goal need to be taken. If it is not the wish of the community to maintain that pronunciation, then it is also not something that should realistically be expected or worked towards. I have added the following text: “If the desire of the community is to maintain specific speech patterns present in older L1 speakers, then from a practical standpoint, it seems that additional resources need to be committed to this achieve this goal.”

 You conclude that intervention and training is needed here and in the plain language summary. But the advanced C-Level speakers did produce this contrast without having had special training. It could therefore be argued that more language exposure and use is required instead.

**Response:** True, but realistically, that’s not possible given the present situation in Lower Sorbian. Few L1 speakers remain and while I agree that significant exposure to L1 speech with such a contrast would/could facilitate learning, that simply isn’t the situation in the present context. If the goal of the Sorbian community is to maintain this contrast (and others), then specific resources would need to be mobilized as it is not realistic or even possible that children or adults would receive significant input stimuli from L1 speakers or even L2 speakers who produce the target contrasts reliably.
